# Supplementary material for: Charge-Discharge Characteristics of Textile Energy Storage Devices Having Different PEDOT:PSS Ratios and Conductive Yarns Configuration
Source: Polymers (Basel). 2019 Feb 16;11(2):345. doi: 10.3390/polym11020345 (PMC6419215; doi:10.3390/polym11020345)
Supplement: Supplementary file 1 [file polymers-11-00345-s001.pdf]

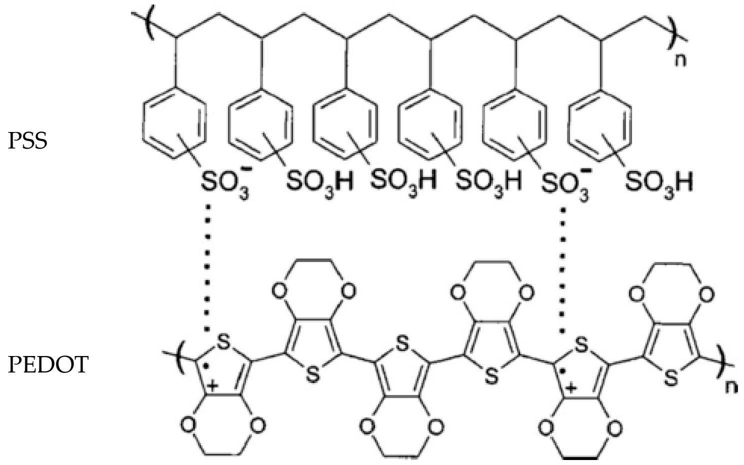

Supplementary Figure 1: Chemical structure of PEDOT:PSS [19]

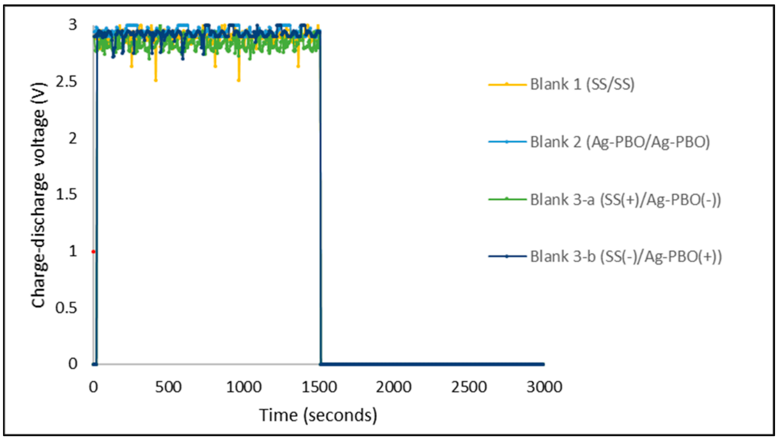

Supplementary Figure 2: Charge-discharge characteristics of blank devices (without PEDOT:PSS) using various electrodes (SS/SS, Ag-PBO/Ag-PBO, SS(+)/Ag-PBO(-), and SS(-)/Ag-PBO(+)), each charged at 3 volt.
